# Sonic Hedgehog Protein Is Decreased and Penile Morphology Is Altered in Prostatectomy and Diabetic Patients

**DOI:** 10.1371/journal.pone.0070985

**Published:** 2013-08-14

**Authors:** Nicholas L. Angeloni, Christopher W. Bond, Kevin T. McVary, Carol A. Podlasek

**Affiliations:** 1 Department of Urology, Northwestern University, Feinberg School of Medicine, Chicago, Illinois, United States of America; 2 Division of Urology, Southern Illinois University School of Medicine, Springfield, Illinois, United States of America; Clermont Université, France

## Abstract

Erectile dysfunction (ED) is a debilitating medical condition and current treatments are ineffective in patients with cavernous nerve (CN) injury, due to penile remodeling and apoptosis. A critical regulator of penile smooth muscle and apoptosis is the secreted protein sonic hedgehog (SHH). SHH protein is decreased in rat prostatectomy and diabetic ED models, SHH inhibition in the penis induces apoptosis and ED, and SHH treatment at the time of CN injury suppresses smooth muscle apoptosis and promotes regeneration of erectile function. Thus SHH treatment has significant translational potential as an ED therapy if similar mechanisms underlie ED development in patients. In this study we quantify SHH protein and morphological changes in corpora cavernosal tissue of control, prostatectomy and diabetic patients and hypothesize that decreased SHH protein is an underlying cause of ED development in prostatectomy and diabetic patients. Our results show significantly decreased SHH protein in prostatectomy and diabetic penis. Morphological remodelling of the penis, including significantly increased apoptotic index and decreased smooth muscle/collagen ratio, accompanies declining SHH. SHH signaling is active in human penis and is altered in a parallel manner to previous observations in the rat. These results suggest that SHH has significant potential to be developed as an ED therapy in prostatectomy and diabetic patients. The increased apoptotic index long after initial injury is suggestive of ongoing remodeling that may be clinically manipulatable.

## Introduction

Erectile dysfunction (ED), the inability to achieve or maintain erection, is a serious medical condition that has high impact on quality of life in 52% of men aged 40 to 70 [1] and 22% of men under 40 [2]. Men at high risk for ED include aging, those with diabetes and prostatectomy or radiation treatment for prostate cancer. In each of these conditions, an underlying cause of ED development is damage to the cavernous nerve (CN), a parasympathetic, peripheral nerve that provides innervation to the penis. Denervation causes remodeling of penile architecture including apoptosis of corpora cavernosal smooth muscle [3] and fibrosis, making the erectile tissue less able to respond to normal signaling paradigms. Current treatments aim to increase smooth muscle relaxation, however this strategy is frustrated by smooth muscle loss due to apoptosis [4]. Thus phosphodiesterase type 5 inhibitors (PDE-5i) are ineffective in 16–82% of prostatectomy and in 56–59% of diabetic patients [5]–[6]. Because of the loss of the critical smooth muscle, new therapies that prevent such architectural changes of the penis are essential. These clinical studies and data from rodent models of ED prompt questions about how smooth muscle apoptosis is regulated in the penis.

In rats, the secreted protein Sonic Hedgehog (SHH) plays a prominent role in establishing and maintaining penile morphology necessary for erection [7]–[9]. The penis is unique in that it undergoes most of its differentiation after birth and SHH functions during the postnatal period to establish corpora cavernosal sinuses [8], which allow blood to flow into the erectile tissue during erection. In the adult, SHH maintains the sinusoid architecture that it initially helped to establish [8], by regulating smooth muscle apoptosis. With SHH inhibition, a 12-fold increase in apoptosis occurs in penile smooth muscle that causes ED [9], [7]. Inhibition of SHH signaling occurs in the penis of two commonly studied ED models, the BB/WOR diabetic and the CN injured Sprague Dawley (prostatectomy model) rat [8], [9]. The decreasing SHH protein, which occurs in response to CN injury, becomes a driving force behind morphological remodeling and ED induction. Whether similar signaling mechanisms underlie development of ED in humans is unknown. Its potential role in ED prevention strategies has significant implications since SHH treatment of CN injured rats via Affi-Gel beads or by peptide amphiphile nanofiber hydrogels suppresses penile apoptosis and improves erectile function by ∼60% at 6 weeks after injury [9]–[12]. We propose that decreased SHH protein is an underlying cause of adverse penile remodeling and resultant ED, in patients with diabetes or following prostatectomy.

To test this hypothesis we examined corpora cavernosal tissue and quantified changes in SHH signaling and morphology from patient’s with Peyronie’s (control), diabetics and following prostatectomy. Parallel morphological changes and decreased SHH protein were observed in ED patients as in animal models. Since localized SHH treatment is effective in suppressing neuropathy-induced apoptosis and improves erectile function by ∼60 in rats [11], SHH treatment may offer opportunities to prevent morphological changes and abort a pathway to certain ED in prostatectomy and diabetic patients. This study is highly significant since it is the first to show that morphological changes that underlie ED development in patients is identical to that observed in rat ED models. Clinical translation of this study into an effective ED therapy is the next logical avenue for this research.

## Materials and Methods

### Patient Tissue

Corpora cavernosal tissue was obtained from 54 patients who underwent penile prosthesis implantation at Northwestern Memorial Hospital. 21 of the patients had previous prostatectomy surgery and 22 patients had diabetes. Tissue from 13 control patients was assayed, including 12 patients that underwent corrective surgery for Peyronie’s disease and one patient with repair of penile fracture. Peyronie’s disease is a condition of the penis characterized by the alteration in the appearance and cellularity of collagen within the tunica albuginea, which becomes fibrotic as the disease progresses. While Peyronie’s patients have intromission difficulty, the underlying defect is tunical in origin so the lacunar tissue remains fundamentally normal [13]. Thus Peyronie’s tissue is the best available control other than cadaver tissue and corporal tissue was obtained from a region away from the involved tunica. Exclusion criteria included male patients over 18 years of age. Corpora cavernosal tissues were frozen in liquid nitrogen or fixed in 4% paraformaldehyde overnight at 4°C prior to paraffin embedding. The complete study protocol, including experimental procedures, was approved by the institutional review board of Northwestern University and written informed consent was obtained from all patients. For the prostatectomy patients it had been 1–17 years since surgery with an average of 6 years. For the diabetic patients, the onset of diabetes was between 7 and 24 years with an average of 12 years. IIEF scores are listed in Table 1.

**Table 1:** ED severity as measured by IIEF in control, prostatectomy and diabetic patients.

Control tissues		
ED severity	Number of patients	IIEF
No ED	4	26–30
Mild ED	1	22–25
Moderate ED	1	17–21
Severe ED	7	≤16
**Prostatectomy tissues**		
**ED severity**	**Number of patients**	**IIEF**
No ED	1	26–30
Mild ED	0	22–25
Moderate ED	1	17–21
Severe ED	19	≤16
**Diabetic tissues**		
**ED severity**	**Number of patients**	**IIED**
No ED	1	26–30
Mild ED	0	22–25
Moderate ED	0	17–21
Severe ED	20	≤16

### Animals

Sprague-Dawley rats postnatal day 115–120 (P115–P120) were obtained from Charles River. The study was carried out in strict accordance with the recommendations in the Guide for the Care and Use of Laboratory Animals of the National Institutes of Health. The animal care protocol was approved by the IACUC committee of Northwestern University and animals were cared for in accordance with institutional IACUC approval.

### Sildenafil Treatment

Pelvic ganglia (PG)/CN were exposed and microforceps (size 0.02×0.06 mm) were used to crush the CN bilaterally for 30 seconds as described previously [11], [14]–[15]. Rats were injected with 60 mg/kg/day sildenafil citrate or DMSO (control) for 7 (n = 6) or 14 days (n = 8) prior to sacrificing the rats and freezing the corpora cavernosal tissue. This concentration of sildenafil is commonly used in rats in the literature [16].

### In Situ

*In situ* was performed as previously described [17], [7], [8] on corpora cavernosal tissue from control (n = 6), prostatectomy (n = 6) and diabetic (n = 5) patients, assaying for *Shh* RNA. A *Shh* RNA probe was obtained from Andrew McMahon [18].

### Immunohistochemical Analysis (IHC)

IHC was performed using the LSAB+ peroxidase kit (DAKO) or fluorescently as previously described [11] assaying for SHH (1/50, Santa Cruz, sc-1194), PTCH1 (1/50, Santa Cruz), BDNF (1/50, R&D), α-ACTIN (1/100, Sigma) and CD31 (Cell Signaling). Fluorescent secondary antibodies were Alexa Fluor 594 chicken anti-goat and donkey anti-mouse 350 (1/150, Life Technologies).

### Trichrome

Trichrome stain was performed as described previously [19] on corpora cavernosal tissue from control (n = 6), prostatectomy (n = 8) and diabetic (n = 6) patients by Image J analysis (Image J version 1.45 s, down load date 5/22/2012). After background subtraction, the total area of blue (collagen) and of red (smooth muscle) were selected and quantified in trichrome photos. Total area of blue/collagen and of red/smooth muscle were measured in 20 photos (100×magnification) taken randomly in corpora cavernosal tissue from each patient (5 photos per section and 4 sections per patient).

### Apoptotic Index

TUNEL was performed using the Apoptag kit (Millipore) on control (n = 5), prostatectomy (n = 8) and diabetic (n = 7) corpora cavernosa. All cells were stained with DAPI (0.005 µg/mL). The number of apoptotic cells/all cells was counted in five regions per section and five sections per patient.

### Western

Corpora cavernosal tissue from each patient was homogenized as outlined previously [9]. 100 µg protein were separated via electrophoresis using a 10% polyacrylamide gel and transferred to a nitrocellulose membrane (Bio-Rad) using a mini Trans-Blot electrophoretic transfer cell (Bio-Rad) for 2 hours. Membranes were blocked with 5% nonfat skim milk in PBS-Tween and were incubated with either 1/50 SHH (Santa Cruz, sc-1194) 1/50,000 α-ACTIN (Sigma), 1/50,000 β-ACTIN (Sigma), or 1/1,000 GAPDH (Cell Signaling), overnight at 4°C. Secondary antibodies were horseradish peroxidase conjugated 1/40,000 donkey anti-goat, 1/80,000 chicken anti-mouse, or 1/2,000 chicken anti-rabbit (Santa Cruz). Protein bands were visualized using ECL detection reagent (GE Healthcare) and were exposed to Hyperfilm (GE Healthcare). Bands were quantified using Kodak ID software (Rochester, NY). Quantification was performed by determining the ratio of SHH/β-ACTIN, SHH/GAPDH and α-ACTIN/GAPDH. Samples were run in duplicate and the results averaged.

### Statistics

Where comparisons of three groups were made ANOVA with a Dunnett’s posthoc test was performed. Where two groups were compared, a t-test was performed and the results were reported ± the standard error of the mean (SEM). Results were considered significantly different if p≤0.05.

## Results

### *Shh* RNA Localization by *in situ*

**Figure 1:**
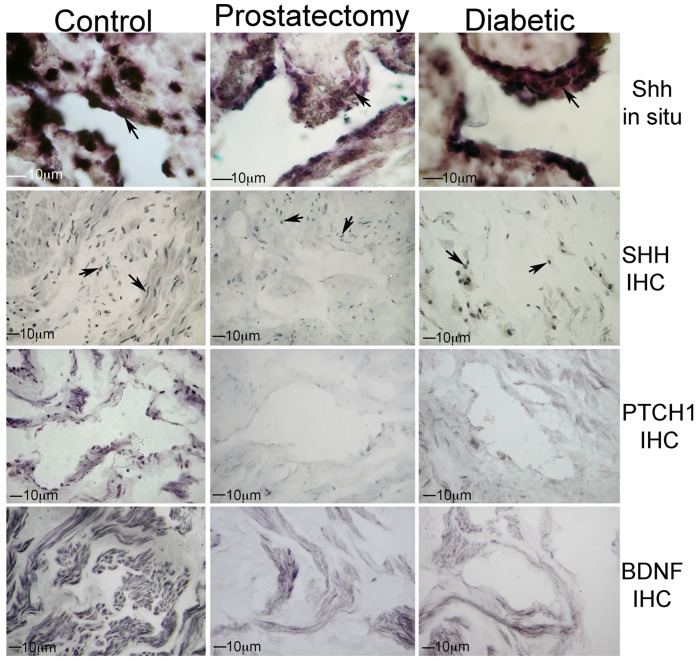
*In situ* for *Shh* RNA and IHC for SHH protein, were performed on corpora cavernosal tissue from control, prostatectomy, and diabetic patients. *Shh* RNA and SHH protein are present in smooth muscle and the localization was unchanged in prostatectomy and diabetic penis. SHH protein appears decreased by visual observation in prostatectomy and diabetic penis. PTCH1 (SHH receptor) and BDNF (SHH target in the rat) were also decreased in prostatectomy and diabetic penis. Arrows indicate staining. 100–400× magnification.

*Shh* RNA is localized in smooth muscle of control, prostatectomy and diabetic patients (Figure 1), indicating that *Shh* is synthesized in penile smooth muscle as observed previously in rat ED models. *Shh* was not identified in the endothelium. There was no change in *Shh* localization in prostatectomy and diabetic tissues (Figure 1).

### SHH Protein Localization by IHC

SHH protein was abundantly expressed in control penis (Figure 1). Localization was unaltered in the prostatectomy and diabetic, however the abundance appeared decreased by visual observation (Figure 1). Patched (PTCH1, SHH receptor) and brain derived neurotrophic factor (BDNF, SHH target in the rat) were identified in control penis and appear to decrease in prostatectomy and diabetic tissues (Figure 1). We recently showed SHH regulation of BDNF in rat penis and CN [20]. Thus the mechanism of how SHH maintains penile morphology and erectile function in patients may involve BDNF.

**Figure 2:**
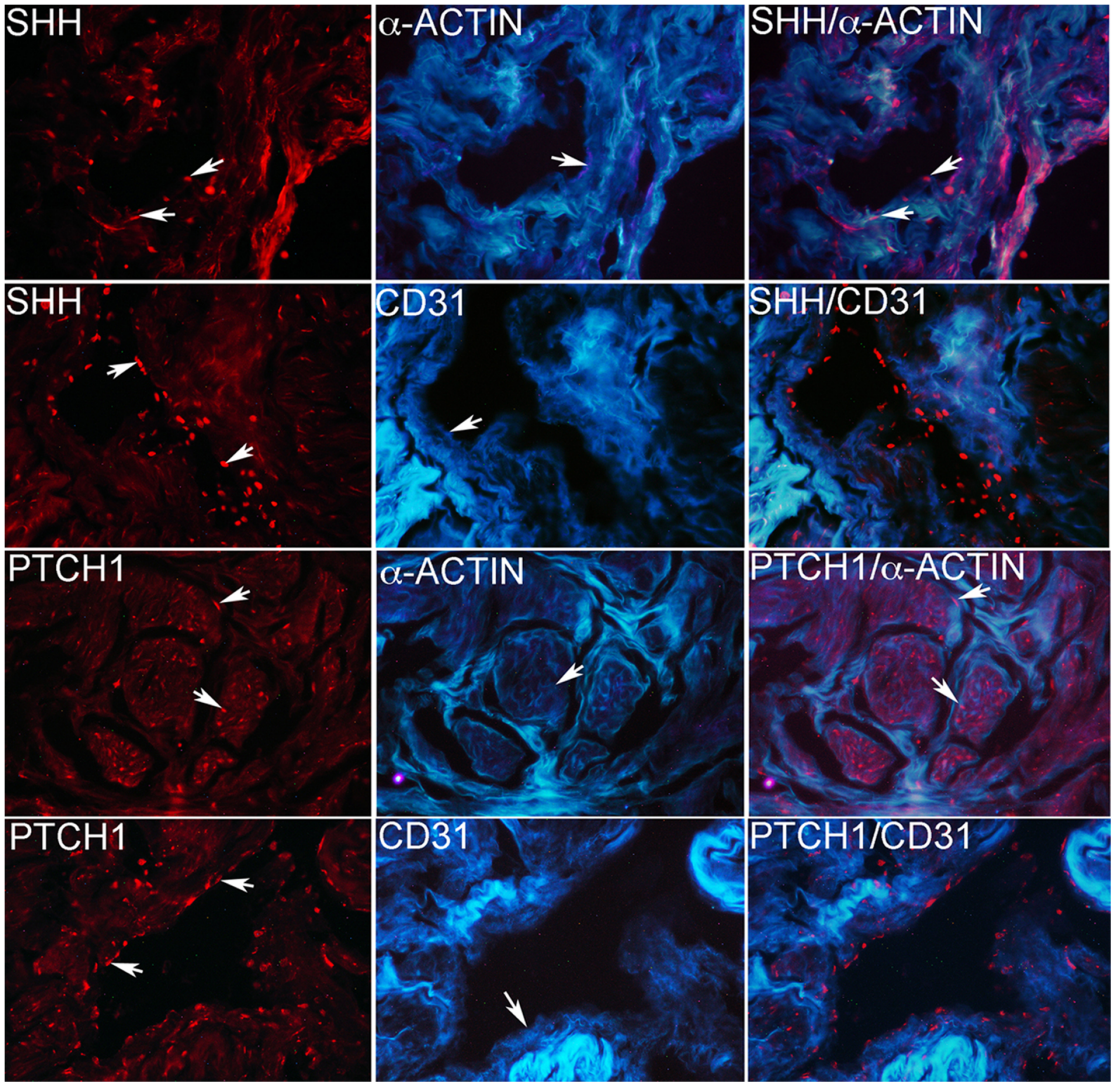
IHC analysis showing dual SHH/α-ACTIN, SHH/CD31, PTCH1/α-ACTIN, and PTCH1/CD31. SHH and PTCH1 co-localize with α-ACTIN in smooth muscle but were not present in the endothelium. Light blue staining is unavoidable auto-fluorescence present in human penis and dark bluish-purple represents α-ACTIN and CD31 proteins. Arrows indicate staining and pink coloration represents co-localization. 250×magnification.

Similar to observations in the rat SHH and PTCH1 co-localize with α-ACTIN in human smooth muscle (Figure 2). Light blue coloration is unavoidable auto-fluorescence present in human penis.

### Trichrome

**Figure 3:**
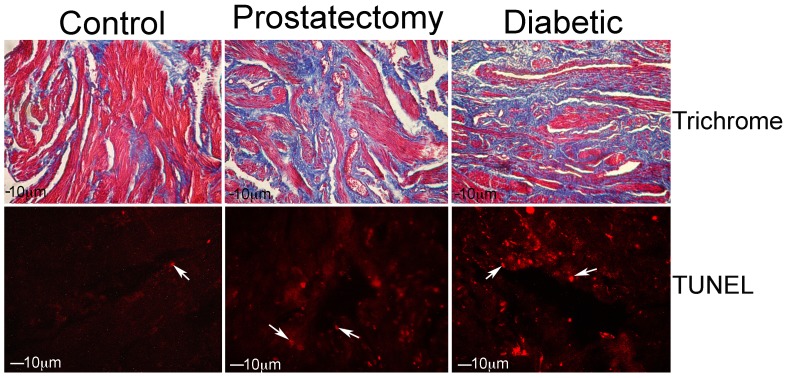
Trichrome and TUNEL were performed on corpora cavernosal tissue from control, prostatectomy, and diabetic patients. The smooth muscle/collagen ratio decreased 25% in prostatectomy and 28% in diabetic penis (p = 0.0338). The apoptotic index increased 22% in prostatectomy and 25% in diabetic penis (p = 0.0141). Arrows indicate staining. 100–400× magnification.

Smooth muscle (red) and collagen (blue) were abundant in controls (n = 6) however the smooth muscle/collagen ratio decreased 25% in prostatectomy (n = 8) and 28% in diabetic (n = 6) patients by Image J analysis (Control: 1.30±0.13, Prostatectomy: 0.97±0.07, Diabetic: 0.94±0.10, p = 0.0338, Figure 3).

### Quantification of the Apoptotic Index

**Figure 4:**
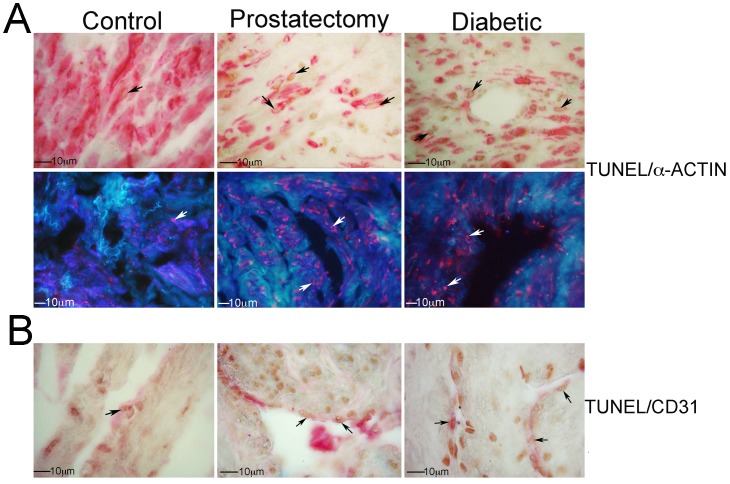
(A) Dual TUNEL/α-ACTIN IHC was performed using colorimetric and fluorescent techniques. Apoptosis was barely detectable in control penile smooth muscle. Apoptosis was abundant in prostatectomy and diabetic corpora cavernosal tissue and almost all apoptosis observed co-localized with α-ACTIN in penile smooth muscle. TUNEL appears in brown (diaminobenzidine, DAB, top) and red (bottom). α-ACTIN appears in red (top) and bluish-purple (bottom). Light blue staining is unavoidable auto-fluorescence present in human penis. Arrows indicate co-localization (shown in pink on bottom). 400× magnification. (B) TUNEL/CD31 IHC shows that apoptosis is also occurring at a very low level in the endothelium. TUNEL appears in brown (DAB) and CD31 appears in red. Arrows indicate co-localization. 400× magnification. Fluorescent co-localization for TUNEL/CD31 was not possible due to the low abundance of endothelium and the high auto-fluorescence present in human penile tissue.

Apoptotic index was quantified in control (n = 5), prostatectomy (n = 8) and diabetic (n = 7) corpora. Apoptosis was identified at low abundance in control penis and was increased 22% in prostatectomy and 25% in diabetic penis (Control = 0.34±0.02, Prostatectomy = 0.44±0.01, Diabetic = 0.45±0.04, p = 0.0141) penis (Figure 3). Apoptosis was abundant in smooth muscle as confirmed by dual TUNEL/α-ACTIN IHC and was barely detectable in endothelium by TUNEL/CD31 IHC in prostatectomy and diabetic penis (Figure 4).

### Quantification of SHH and α-ACTIN by Western

**Figure 5:**
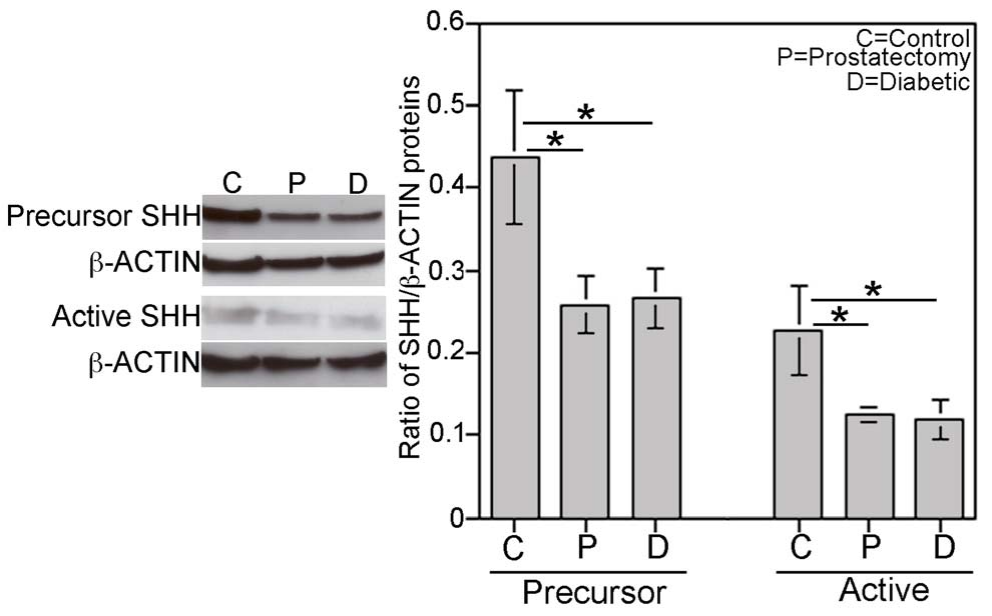
Quantification of precursor and active SHH protein by western analysis of corpora cavernosal tissue from control, prostatectomy and diabetic patients. Precursor SHH protein significantly decreased 41% in prostatectomy and 39% in diabetic patients (p = 0.0312). Active SHH protein significantly decreased 46% in prostatectomy and 48% in diabetic patients in comparison to controls (p = 0.0222). Asterisks indicate significance. Where error bars do not appear they are too small to be shown on the graph.

**Figure 6:**
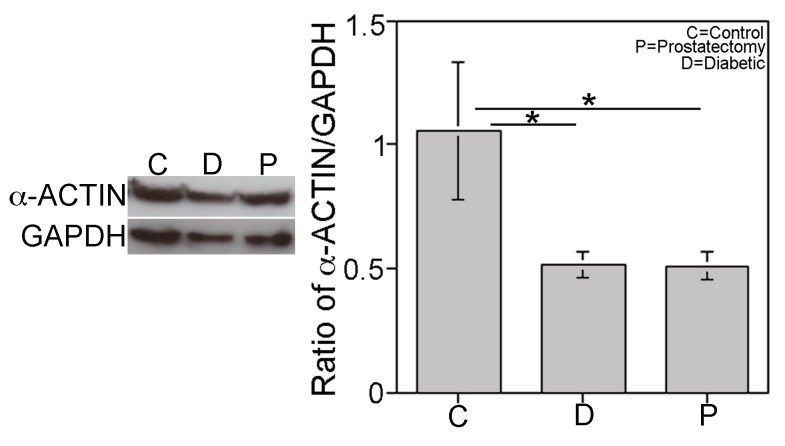
Quantification of α-ACTIN by western analysis of corpora cavernosal tissue from control, prostatectomy and diabetic patients. α-ACTIN was significantly decreased 52% in prostatectomy and 51% in diabetic (p = 0.005) corpora cavernosa.

Precursor and active SHH protein were quantified by Western in control, prostatectomy and diabetic corpora cavernosa. Precursor SHH protein decreased 41% in prostatectomy (n = 17) and 39% in diabetic (n = 18) penis in comparison to controls (n = 10, p = 0.0312, Figure 5). Active SHH protein was significantly decreased 46% in prostatectomy (n = 19) and 48% in diabetic corpora (n = 19, Figure 5) in comparison to controls (n = 9, p = 0.0222). α-ACTIN/GAPDH was used to quantify smooth muscle, which was significantly decreased 52% in prostatectomy (p = 0.005, n = 21) and 51% in diabetic patients (p = 0.005, n = 22) relative to controls (n = 11, Figure 6).

### Quantification of SHH Protein by Western in Sildenafil Citrate Treated Rats

**Figure 7:**
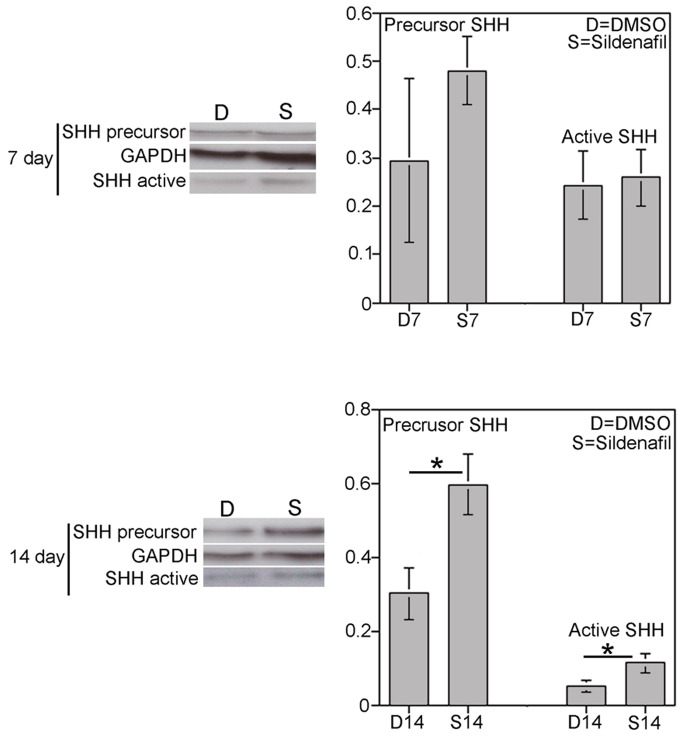
Quantification of precursor and active SHH protein by western analysis of rats that underwent bilateral CN crush and sildenafil or DMSO treatment for 7 or 14 days. Precursor and active SHH proteins were significantly increased 50% and 57% (p = 0.02 and 0.04) after 14 days of sildenafil treatment.

Precursor and active SHH protein were quantified by Western in corpora cavernosal tissue of rats that underwent CN crush and either sildenafil (n = 3,4) or DMSO (control, n = 3,4) treatment for 7 or 14 days. Precursor and active SHH protein did not change with sildenafil treatment for 7 days (p-value = 0.18 and 0.44, respectively, n = 3,3, Figure 7). However, precursor and active SHH protein significantly increased 50% and 57% at 14 days of sildenafil treatment (p-value = 0.02 and 0.04, n = 4, 4, Figure 7). This result is significant because ED patients are commonly prescribed PDE5i following prostate cancer surgery which in addition to promoting smooth muscle relaxation via the nitric oxide synthase/cGMP pathway may also improve penile morphology and thus erectile function through a SHH dependent mechanism.

## Discussion

In this study we identify active SHH signaling in human corpora cavernosal tissue. This is the first examination of the SHH pathway in adult human penis and assessment of its importance to disease development in ED patients. Both precursor and active SHH protein, were significantly decreased in prostatectomy and diabetic tissues. This is important because in animal models inhibiting SHH increases smooth muscle apoptosis, causing remodeling of penile architecture and loss of erectile function. In patients, a ∼50% reduction in smooth muscle accompanied declining SHH protein, suggesting that similar remodeling occurs in ED patients. The apoptotic index significantly increased 22% in prostatectomy and 25% in diabetic tissues. In these men the time since prostatectomy ranged between 1–17 years with an average of 6 years and the onset of diabetes occurred between 7–24 years previously with an average duration of 12 years. The increased apoptotic index is substantial and surprising given the duration since the original nerve insult (prostatectomy or diabetic neuropathy) and is suggestive of an ongoing remodeling process that may be clinically manipulatable long after the injury. In the rat, CN resection [3] or crush [12] results in apoptosis primarily in the first week after injury, with a low level continuing until 14 days for crushed [12] and 4 weeks for the resected group [3]. In the rat there is some degree of CN regeneration that occurs over time, which might account for decreased apoptosis after the initial injury. In contrast, remodeling of human penis appears to be an ongoing process once initiated. This emphasizes the importance of early preventative strategies in ED patients.

Identification of decreased SHH protein in ED patients is highly significant for development of future therapies. We previously showed that SHH treatment of CN injured rat penis suppresses apoptosis and improves erectile function by ∼60% at 6 weeks after injury [10], [11]. Since similar changes in SHH signaling and penile morphology occur in prostatectomy and diabetic patients as in rat ED models, there is substantial potential for development of SHH as a therapy to preserve penile architecture after neurological insult. Peripheral nerves have regenerative potential and some degree of regeneration does occur after prostatectomy. A significant sequela of this neuropathy is the down stream penile morphological changes that degrade erectile function. Smooth muscle is less abundant and the lacunar tissues become non-responsive to NO-cGMP signaling once innervation is re-established, thus ED is unavoidable. Upregulating SHH protein rather than RNA is critical for preserving penile morphology since we previously observed a block in SHH protein processing in CN injured rats [9]. We have developed a novel type of delivery vehicle for SHH protein to the rat penis using a peptide amphiphile nanofiber hydrogel (PA) [10]. This nanotechnology delivery of SHH effectively suppresses penile apoptosis and improves erectile function after CN injury [10], [11]. The SHH PA is biodegradable and may be developed for local SHH delivery to the penis of ED patients to preserve penile architecture. We’ve shown in previous studies that morphological changes that occur in the rat penis in response to SHH inhibition are reversible [9], therefore clinical application of the SHH PA is the next logical step.

PDE5i may be a way to increase SHH in the penis. Several recent studies in animal models and humans demonstrated anti-apoptotic and anti-fibrotic effects of PDE-5i following CN injury [21]–[25]. Treatment of CN crushed rats with udenafil, a PDE5i elevated SHH protein in the penis of rats with eight weeks of treatment [26]. This is significant because many of the patients in this study, had PDE5i use prior to prosthesis implantation. Thus we performed a controlled study in rats examining the effects of sildenafil on SHH abundance in the penis. Precursor and active SHH protein were significantly increased 50 and 57% in response to sildenafil treatment for 14 days. However clinical studies show that PDE5i treatment fails in many patients. This suggests that PDE5i may only transiently increase SHH or be insufficient for full recovery. PDE5i may also not increase SHH enough in the immediate post-injury period to suppress long term morphological remodeling. Thus a direct and controlled application of SHH to the corpora is a more effective strategy.

A limitation of this study may be the use of Peyronie’s tissues as controls. While Peyronie’s patients have intromission difficulty, the underlying defect is tunical in origin so the lacunar tissue remains fundamentally normal. Thus Peyronie’s tissue is the best available control other than cadaver tissue and corporal tissue was obtained from a region away from the involved tunica. Another limitation of this study is that control patients with ED of lesser severity were not examined since it was not possible to obtain tissue. This was also the case with men who underwent radical prostatectomy who recovered erectile function. The prostatectomy tissue examined may not be fully representative of the clinical situation since the tissues were from patients with severe ED.

### Conclusions

In conclusion, parallel morphological changes and decreased SHH protein were observed in animal models of ED and in ED patients that have had a prostatectomy or are diabetic. Since localized SHH treatment is effective in suppressing neuropathy-induced apoptosis and improves erectile function in rat models, SHH treatment has the potential to be similarly effective in prostatectomy and diabetic patients.
